# Evaluation and Reduction of CRISPR Off-Target Cleavage Events

**DOI:** 10.1089/nat.2019.0790

**Published:** 2019-08-06

**Authors:** Christopher A. Vakulskas, Mark A. Behlke

**Affiliations:** Integrated DNA Technologies, Inc., Coralville, Iowa.

**Keywords:** CRISPR/Cas9, hematopoietic stem cells, gene editing, AAV

## Abstract

Introduction of CRISPR/Cas9 methods (clustered regularly interspaced short palindromic repeats, CRISPR-associated protein 9) have led to a huge surge in the use of precision genome editing for research applications. Translational medical efforts are likewise rapidly progressing, and Phase I clinical trials using these techniques have already started. As with any new technology that is applied to medical therapeutics, risks must be carefully defined and steps taken to mitigate side effects wherever possible. Effective methods are now available that permit identification of off-target cleavage events, a major class of potential side effects seen in mammalian genome editing. Off-target prediction algorithms are improving and have utility, but are insufficient to use alone. Empiric methods to define the off-target profile must also be used. Once defined, the frequency of off-target cleavage can be minimized using methods that limit the duration of exposure of the genome to the active genome editing complex, for example, using the ribonucleoprotein (RNP) approach. In addition, Cas9 mutants have been developed that markedly reduce the rate of off-target cleavage compared to the wild-type enzyme. Use of these new tools should become standard practice for medical applications.

## Introduction

The field of genome editing is revolutionizing modern medicine by allowing the targeted alteration of DNA in live cells and animals with the goal that previously untreatable genetic disorders might be cured. Systems that alter genomic DNA are not new, however, recent introduction of CRISPR (clustered regularly interspaced short palindromic repeats) methods that use the Cas9 (CRISPR-associated protein 9) nuclease from *Streptococcus pyogenes* have gained widespread adoption and led to a level of excitement heretofore not seen in the field. One concern that argues caution when applying CRISPR genome editing (or any other method that results in permanent genetic changes in humans) is the risk that off-target cleavage may occur at unintended sites in the genome and lead to adverse effects. Preclinical development requires study and mitigation of off-target risks for any genome editing treatment before testing in humans. Ideally, the sites/sequences that are selected for use will have low homology to the rest of the genome and be intrinsically “low risk.” However, predicting real off-target sites computationally has proved to be extremely challenging. It is possible to empirically determine the identity of real off-target cleavage sites, but such studies require access to advanced next-generation sequencing (NGS) and bioinformatics technologies, and there is no certainty that all off-target risks are identified by any given method. This is further complicated in medical applications where every patient has a unique genome and therefore a unique off-target risk profile. Ultimately, better Cas9 cleavage prediction algorithms will reduce reliance on complex empiric tools by enabling better original site selection. Nevertheless, the actual genome editing site used will often be defined by the location of a disease-causing mutation and therefore cannot be changed due to the possibility of off-target risks at that site. In this case, specificity of the genome editing machinery must be improved. Progress has been made in developing methods that improve specificity of the CRISPR-Cas9 system. For example, off-target activity varies with the method of delivery of the genome editing components to cells. The CRISPR guide RNAs (gRNAs) can be chemically modified in ways that reduce their potential to target sites with imperfect complementarity. Finally, mutant Cas9 enzymes have been developed that intrinsically have higher specificity.

## How We Got Here: Meganucleases, Zinc Fingers, Transcription Activator-Like Effector Nucleases, and CRISPR-Cas9

The field of genome editing or “gene targeting” originated with successful homologous recombination in mammalian cells in the absence of a double-stranded DNA (dsDNA) break. In these early experiments, investigators were able to achieve gene replacement using a dsDNA donor that contained a DNA sequence of interest flanked by “homology arms” that aligned with complementary sites in the genome and led to homologous recombination, although at low frequency (typically 10^−7^ to 10^−6^) [1,2]. It was later discovered that site-specific dsDNA breaks (DSBs) served as potent substrates for homologous recombination (showing as much as a 10^5^-fold increase in efficiency) and subsequent nuclease-dependent gene targeting methods are all based on this principle [3–5].

The first reported gene targeting experiments with DSBs were performed with the rare-cutting *I-S*ceI “meganuclease,” which recognizes an 18-bp restriction site [3,4]. While meganucleases can be used to introduce DSBs into mammalian cells with high efficiency, changing their cut-site sequence specificity to be useful in sites of interest can be extremely challenging [6]. A more malleable solution presented itself with the discovery that the *Fok*I restriction enzyme could be split into separate and functional DNA-binding and DNA-cleavage domains [7,8]. This allowed for the creation of zinc-finger nucleases (ZFNs), which consist of the *Fok*I DNA-cleavage domain fused to three or more zinc-finger protein domains [9]. Each zinc-finger domain interacts with specific trinucleotide motifs, which allows for the creation of custom DNA recognition domains through protein engineering [10–12]. While ZFNs have been used successfully to perform genome editing in both animals and plants [13–17], these engineered proteins can have significant off-target cleavage activity, cytotoxicity, and difficulty in obtaining the desired DNA recognition module [17]. Many of these problems were resolved by replacing zinc-finger domains with a different protein module having DNA sequence-specific binding: transcription activator-like effector nucleases (TALENs) from *Xanthomonas* bacteria. This system has lower toxicity in human cells and possesses a more robust and easily programmable DNA-binding domain [18]. While both ZFNs and TALENs are currently in clinical development, both systems require a significant protein engineering effort that serves as a barrier to widespread adoption and routine use.

The first widespread example of RNA-guided genome editing comes from the study of group II introns or “targetrons” that utilize a ribozyme and reverse transcriptase to cleave targeted DNA through a process called retrohoming [19]. These systems are widely used in prokaryotes where they function with both high efficiency and specificity. However, these systems are poorly adapted to eukaryotic species where they function inefficiently [20]. An elegant alternative to ZFNs, TALENs, and targetrons presented itself in 2012 with characterization of the RNA-guided DNA endonuclease CRISPR-Cas9 system from *S. pyogenes* [21]. CRISPR-Cas9 targeting is directed by a gRNA complex that is formed by hybridization of a nontargeting universal trans-activating CRISPR RNA (tracrRNA) and a targeting CRISPR RNA (crRNA). The Cas9 nuclease binds directly to the gRNA to form a ribonucleoprotein (RNP) complex that cleaves dsDNA at sites defined by a 20-nt sequence in the targeting region of the crRNA (the protospacer) only when adjacent to a specific sequence in the genomic DNA that is not included in the protospacer RNA. In the case of SpCas9, the required protospacer-adjacent motif (PAM site) is “NGG.” Unlike ZFNs and TALENS, the CRISPR-Cas9 system can be directed to cleave 3-bases upstream from any NGG sequence simply by supplying an appropriate gRNA without the need for any site-specific protein engineering. It was subsequently demonstrated that CRISPR-Cas9 could be delivered into live human cells and other organisms to facilitate highly efficient genome editing [22–25]. The relative simplicity and low-cost nature of CRISPR-Cas9 as a genome editing tool led to rapid adoption of this technology for both basic research in academic laboratory settings and for therapeutic genome editing. Before introduction of CRISPR-Cas9 as a genome editing solution, the technological and cost barriers associated with ZFNs and TALENs had largely relegated genome editing technologies to the most well-funded and patient academic and industrial users.

RNA-guided cleavage by RNA endonucleases in living cells or “RNA editing” has been described for several recently discovered CRISPR systems. The notion of RNA editing, pioneered through discovery and characterization of group I introns [26], may play important future roles in medical diagnostics, but will not be discussed further in this opinion piece that focuses on DNA-targeting genome editors [27].

## Predicting Specificity of CRISPR-Cas9 gRNA Sites in Mammalian Cells

Native CRISPR-Cas9 systems function in bacteria as part of a primitive adaptive immune system that protects genomic integrity from invading foreign DNA [21]. To provide an adequate defense, CRISPR-Cas9 systems must be able to recognize highly variable genetic elements from phages or other foreign invaders and rapidly adapt to these new threats. Bacterial genomes are much smaller than that of eukaryotes, so Cas9 has evolved in the absence of selection pressure to develop specificity sufficient for unique targeting in mammalian genomes that are 1,000-fold larger. Multiple independent groups have reported that Cas9 cleavage can occur at sites that differ from the intended on-target site at three or more positions within the protospacer or at a single position within the PAM [28–32]. Off-target effects are an expected risk for any therapy or experimental treatment where foreign products are introduced into human cells. Off-target effects have been observed and well-documented not only for genome editing technologies (CRISPR, meganucleases, ZFNs/TALENs, etc.) [32–34] but also for gene knockdown technologies such as RNA interference (RNAi) and antisense [35,36]. Off-target effects in genome editing pose a serious risk in that both *in vivo* and *ex vivo* clinical therapies will likely require the genetic modification of very large cell populations, thereby increasing the likelihood that even a rare off-target risk might lead to some kind of adverse outcome.

In an ideal setting, off-target effects (OTEs) could be computationally predicted for any given gRNA, so high-specificity genome editing could be achieved simply by careful gRNA selection. Numerous attempts have been made at developing algorithms for gRNAs that predict on-target potency and stratify risk for OTEs [37,38]. While progress has been made in developing predictive models for OTEs by Cas9, existing algorithms do not have sufficient specificity and sensitivity to be a primary tool for risk mitigation of therapeutic genome editing. The first level of homology screening compares the gRNA sequence with the genome and catalogs sites as having one, two, or more bases of mismatch (or insertion/deletion events). However, in actual genome editing, off-target sites with a single base mismatch are sometimes seen, which show no cleavage while other sites with three to four mismatches can show near complete cleavage [32]. Cas9 therefore appears to have additional enzymatic cleavage determinants that are not directly dependent on perfect base pairing between guide and target. DNA-accessibility, as defined by histone binding and higher order chromatin structure, varies widely between cell types and species and can also influence the activity seen at each potential Cas9 target (and off-target) site [39]. Because of these complexities, most OTE predictive algorithms fail to consistently call bonafide OTE sites, and/or are unable to predict relative cleavage efficiencies at the hundreds or thousands of predicted sites found by homology alone. Hence, bioinformatics predictions often either miss real OTE sites or report too many sites to easily study. Analyzing thousands of potential OTE sites is impractical and prohibitively expensive for the average researcher and thus improvements in Cas9 target site predictability are needed.

One recent Cas9 off-target site prediction tool “Elevation” incorporates a machine learning approach, wherein a two-layer regression model is used first to predict the off-target activity of a single mismatch and then secondarily combines predictions for gRNA-target pairs that contain multiple mismatches [40]. Data are aggregated to produce a single off-target summary score for each gRNA, which is derived from each individual off-target score between a given gRNA and its potential target sites in the human genome. Predictions from the Elevation software package were compared head-to-head to that of other existing models using data from two independent genome-wide, unbiased assays. The Elevation package consistently outperformed all other models (CFD, Hsu-Zhang, and CCTop) in predicting off-target activity, by an order of magnitude in some cases, and never performed worse than any of the existing models.

Studies have indicated that DNA accessibility (chromatin structure, bound DNA-binding proteins, and so on) can influence CRISPR-Cas9 cleavage in living cells [39]. During the development of the Elevation package, the authors attempted to apply existing DNase I chromatin accessibility data to Cas9 off-target prediction modeling [40]. These efforts were applied separately with data from individual cell lines or with aggregated data that was averaged together from many different cell types. In general, incorporating these data into Cas9 off-target prediction models did not improve off-target site identification or the accuracy of scoring models. Dramatic differences in chromatin accessibility between different cell types makes incorporating this feature into Cas9 prediction algorithms extremely challenging and, to be practically useful, may require search queries that are tied to a specific cell type.

## Detection of Off-Target Cleavage Events *In Vitro*

The ability to detect all cleavage events from a CRISPR-Cas9 genome editing experiment in cell-free *in vitro* systems has the appeal of eliminating the complications of cell culture and having to efficiently delivery CRISPR reagents into living cells. Unlike live cell methods (see Detection of off-target cleavage events *in vivo* section), some of these techniques do not require an existing reference genome, which means that these studies can be done using DNA from virtually any source. The two most commonly used techniques described are known as selective enrichment and identification of tagged genomic DNA ends by sequencing (SITE-Seq) and circularization for *in vitro* reporting of cleavage effects by sequencing (CIRCLE-seq) [41,42].

The SITE-Seq method subjects purified genomic DNA to *in vitro* Cas9 cleavage using preformed RNP complexes. The cleavage products from this reaction, including both on- and off-target sites are tagged, enriched, sequenced, and mapped to a reference genome. The number of Cas9 off-target sites detected using SITE-Seq increase in a concentration-dependent manner. This method does not require the NGS read depth needed for other techniques such as Digenome-seq [43], which are complicated by high background levels. The CIRCLE-seq method is conceptually similar to SITE-Seq, with some significant procedural variations. With this method, genomic DNA is first sheared and circularized; the remaining linear DNA is degraded before Cas9 cleavage to eliminate or significantly reduce background DNA; this step increases sensitivity and reduces NGS read space wasted on background cleavage events. Cas9 cleavage linearizes the genomic DNA circles, which can then be detected by NGS methods. Methods such as SITE-Seq and CIRCLE-seq will typically find many more cleavage sites than are actually observed in live cells, presumably due to the lack of DNA associated proteins and chromatin structure *in vitro* which limit cleavage *in vivo*.

## Detection of Off-Target Cleavage Events *In Vivo*

A wide variety of techniques have been described to identify DSBs in living cells. An excellent summary and description of these methods and their strengths/weaknesses has been described previously [44]. These techniques can be loosely categorized as those that rely on whole-genome sequencing, the insertion of adapter DNA sequence tags for amplification or enrichment, and those that map translocations by joining Cas9-induced DSBs to natural or artificial DSBs.

One popular method to detect real cleavage sites in live cells following genome editing is genome-wide unbiased identification of DSBS enabled by sequencing (GUIDE-seq) [32]. This procedure uses a synthetic blunt dsDNA tag that is inserted into some fraction of healed DSBs by the nonhomologous end-joining (NHEJ) DNA repair pathway. A short, end-protected 34 bp dsDNA GUIDE-seq “tag” is added to Cas9 genome-editing experiments under conditions where the tag sequence is inserted into DSBs with moderately high efficiency. Genomic DNA from these experiments is randomly sheared, end repair is performed, sequencing adapters are ligated to the ends, and an NGS library is generated that can provide sequence information from both ends of the DSB site. While there are no DSB identification techniques for use in live cells that are “simple” by most experimental standards, GUIDE-seq is one of the more straightforward techniques that is also both relatively inexpensive and widely used in the CRISPR field. All DSB identification protocols have drawbacks, and one reported drawback to GUIDE-seq is that a high level of sequencing read depth is required to overcome issues with high background. The procedure also has been reported to occasionally miss authentic OTE sites that are found with other techniques, such as breaks labeling *in situ* and sequencing (BLISS) [45]. In these cases, one possible explanation for the absence of a known OTE site has been that not all DSBs are repaired with the NHEJ pathway, and that these are genomic loci where the GUIDE-seq tag is not inserted due to the prevalence of some other DNA repair pathway. Nevertheless, in this study GUIDE-seq appears to identify the vast majority of authentic OTE sites discovered with other DSB identification techniques. Sites that are missed with GUIDE-seq might also be a (correctible) consequence of using an imperfect data processing pipeline. For example, many of the gRNA sequences used in published comparative studies targeted repeats and were therefore prone to alignment issues in downstream data processing [32]. Additional comparative studies are needed to further define the limitations of GUIDE-seq and to determine whether absent sites represent no tag insertion at a DSB or if these sites were simply missed by the GUIDE-seq data processing pipeline.

While the GUIDE-seq procedure is an excellent tool for the identification of DSB sites, the relative frequency of different off-target cleavage events identified is only semiquantitative [42]. When using GUIDE-Seq or other NGS-based OTE discovery tools, it is usually better to use simpler methods to quantitatively measure OTE frequencies. It is not realistic to perform whole-genome sequencing with sufficient depth to characterize OTEs, which may be present at frequencies ranging from 100+% to <1% of the on-target site. Hence, approaches that enrich for known OTE sites are used, such as capture-based or amplicon-based NGS methods [46]. Depending on the number of sites studied, amplicon-based NGS can be done using singleplex reactions pooled after amplification or with large multiplex pools. The approach of coupling GUIDE-seq as a discovery tool with multiplex amplicon NGS has been used with good results to characterize OTE frequency for various gRNAs while comparing different delivery methods and the use of wild type (WT) versus improved fidelity Cas9 mutants [47]. The two-step approach to first perform off-target site identification and then secondarily quantitatively measure editing efficiencies allows the best available approach to be used at each step, and it also provides a measure of redundancy and confidence in experimental data as the results from both experiments partially overlap and should be in general agreement.

## Mitigation of Off-Target Risk: RNP Delivery

Many different solutions have been proposed to improve specificity of CRISPR-Cas9 genome editing in human cells, including (1) control of the dose or duration of exposure of the cell to the CRISPR reagents [48], (2) chemical modification of the gRNAs [49], or 3) use of improved fidelity Cas9 mutants [47,50–54]. In an ideal setting, the Cas9 protein and gRNA complex would remain in the nucleus just long enough to achieve maximal editing or homology directed repair (HDR) at the desired locus, after which these reagents would naturally degrade or be eliminated by dilution during mitosis. As a general rule, the longer both Cas9 and a gRNA are present in cells, the more opportunity there is for off-target editing to occur. Plasmid or viral based coexpression of Cas9 and a single guide RNA (sgRNA) typically results in sustained overexpression of these components, which leads to high off-target cleavage events [55]. Use of recombinant Cas9 protein with synthetic gRNAs delivered as an RNP complex presents the CRISPR editing components initially at high concentration, giving high efficiency on-target editing, but in a transient manner, a “fast on, fast off” approach. This method results in much lower off-target editing than expression-based approaches. The RNP delivery method provides a limited time window to achieve good on-target editing as peak Cas9 levels typically occur after just a few hours post delivery. To achieve suitable on-target potency in a short period of time, recombinant forms of Cas9 protein have been engineered with optimized nuclear localization signals (NLS) and other features to maximize nuclear delivery and overall efficiency of editing [56,57]. Furthermore, chemically modified sgRNAs have been optimized to provide the most stable and potent targeting RNA possible, which is particularly important in human primary cells or more nuclease-laden cell types [58]. At present, RNP delivery of WT or mutant (discussed in Mitigation of off-target risk: mutant improved-specificity Cas9 enzymes section) Cas9 complexed with chemically modified sgRNAs represents the best option to perform high-efficiency genome editing while minimizing the risk for unwanted off-target editing [47].

## Mitigation of Off-Target Risk: gRNA Modification

Chemical modification of synthetic gRNAs is necessary for optimal function in mammalian cells [58]. Modification provides relative resistance to nuclease degradation and can also limit their ability to trigger innate immune responses. Modifications shown to improve activity include groups such as 2′-*O*-methyl RNA, 2′-F, or DNA residues, with or without internucleotide phosphorothioate linkages [59–62]. Interestingly, use of other modifications, such as 2′-*O*-methyl-3′-phosphonoacetate, can also reduce off-target effects [49]. Mechanistically, it is thought that this class of modification lowers the binding affinity of the gRNA to the DNA target. At off-target sites, the gRNA:target heteroduplex has mismatches, which additionally lower binding affinity compared to the perfect match target, reducing the likelihood that these sites will be sufficiently stable to trigger cleavage. It is not clear if the observed reductions in off-target cleavage actually result from lower RNP/substrate stability or if it relates more for the ability of the imperfect heteroduplex to trigger structural changes within Cas9 that are needed to attain a fully active conformation. While the precise mechanism by which Cas9 maintains specificity is still poorly understood, recent studies indicate that the noncatalytic REC3 domain provides a proofreading function by recognizing target complementarity and triggering activation of the HNH nuclease domain [52]. Strategically placed chemical modifications that destabilize imperfect duplexes may have the effect of reducing off-target cleavage, while the more stable on-target heteroduplex supports cleavage. The major drawback of this technique is that there appear to be multiple sites within the targeting portion of the Cas9 gRNA that can influence off-target effects and which sites need to be modified may vary with sequence; over-modification of the gRNA reduces on-target cleavage [49]. There is no “one size fits all” set of modifications that gives an ideal combination of reduced off-target editing while retaining high on-target activity. In practice, an investigator may need to empirically test a potentially large number of different modification patterns for each given gRNA sequence to achieve the desired on/off-target editing properties. While not necessarily a straightforward or uniform solution to reducing OTEs, gRNA modification does represent a potentially powerful tool for reducing OTEs in scenarios where other options are limited or unavailable.

## Mitigation of Off-Target Risk: Mutant Improved-Specificity Cas9 Enzymes

The crystal structure for Cas9 complexed with a gRNA and the DNA target/substrate provide a blueprint to introduce mutations that confer improved specificity [63,64]. Researchers using this “rational engineering” approach reasoned that mutation of amino acid residues that served as contact points for Cas9 binding the nucleic acid substrate might lower overall binding affinity and affect off-target cleavage efficiency. Slaymaker *et al.* mutated amino acid residues involved with Cas9 binding the nontargeted DNA strand [50]. It was predicted that, at off-target sites, mismatches between the gRNA and target DNA would lead to additional destabilization of the RNP/substrate complex and result in reduced cleavage efficiency at off-target sites, while perfect-match on-target sites would be unaffected. Many different combinations of alanine substitution mutations were analyzed for off-target editing using gRNAs with known off-target profiles. Mutants were also analyzed using a series of altered gRNAs having single mismatches positioned throughout the targeting protospacer sequence. Several mutants were found that improved Cas9 cleavage specificity and combining three of these mutations had additive effects. Ultimately the authors of this study settled on a set of mutations (K848A, K1003A, R1060A) that together resulted in the lowest level of OTEs while retaining on-target editing in the system studied. The final optimized mutant was called eSpCas9(1.1).

A similar strategy was used by Kleinstiver *et al.* to direct rational mutagenesis to disrupt contact residues between Cas9 and the targeted DNA strand [51]. The resulting optimized mutant, called SpCas9-HF1 (N497A, R661A, Q695A, Q926A), demonstrated improved editing specificity in human cells when paired with a variety of gRNAs. Doudna and colleagues continued this line of investigation and developed another improved specificity Cas9 mutant called “HypaCas9” (N692A, M694A, Q695A, H698A) [52]. Notably, physical studies of Cas9 conformation and substrate affinity demonstrated that the improved fidelity of the new mutant Cas9 did not stem from reduced substrate affinity, as had previously been postulated, but instead arose from suppressing the final transition of Cas9 conformation needed to reach an active state, a process that involved a previously unknown target site proofreading mechanism built into the REC3 domain of SpCas9.

It is important to note that the eSpCas9(1.1), SpCas9-HF1, and HypaCas9 mutants were selected and studied for functional performance (on- and off-target editing) using plasmid-based sustained overexpression of both Cas9 and the gRNA [50,51]. All bear multiple mutations that together confer exceptional increases in on-target specificity. However, others have observed that all these mutants suffer from reduced on-target activity when used in the more transient RNP CRISPR-Cas9 genome-editing method. It may be that multiple mutations had to be incorporated in these variants to fully suppress the high level of off-target activity experienced when using plasmid expression systems, which inherently maximize the off-target problem. For therapeutic applications where use of viral or plasmid vectors is acceptable, these mutants may provide a solution to the off-target problem. However, they do not appear to be suitable for use when a DNA-free RNP approach to genome editing is used [47].

Rather than using intelligent design based on known crystal structure, Vakulskas *et al.* used a bacterial genetic screen to empirically identify Cas9 mutants in a randomly mutated library with improved specificity [47]. In this screen, dual selection pressure was applied in bacteria that required surviving clones that had both reduced off-target activity and high on-target activity. Candidate mutations from this screen were tested in human cells with a fixed quantity of chemically synthesized gRNAs and the best performing mutants were purified and tested in RNP format. The performance of both individual mutations or stacked/combination mutants was examined. The best performing isolate had only a single point mutation (R691A) and displayed increased specificity while maintaining near WT levels of on-target performance with RNP delivery. This newly identified high fidelity mutant is called “HiFi Cas9.” Interestingly, all double or triple mutants tested had reduced on-target activity. The new Cas9 R691A (HiFi Cas9) was demonstrated to function well in an *ex vivo* system to repair the p.E6V mutation in the hemoglobin beta (*HBB*) gene that causes sickle cell anemia. HiFi Cas9 was introduced into human CD34^+^ hematopoietic stem cells as an RNP complex by electroporation along with an adeno-associated virus (AAV) HDR template. The HiFi Cas9 showed a 20-fold reduction in off-target effects compared to WT Cas9, and on-target gene correction rates were similar for both Cas9 forms. Plans are in place to start a Phase 1 clinical trial to treat sickle cell disease using this *ex vivo* protocol using HiFi Cas9 as RNP in the near future.

Using a mutant Cas9 that has been engineered for improved specificity seems to be a prudent choice whenever high-fidelity genome editing is needed, and all medical applications surely fall in this category. Thankfully multiple options exist to choose from. The optimal choice of which version to use will likely vary with the planned delivery method and treatment setting. For the present, HiFi Cas9 seems to be the primary choice if RNP methods are planned [47]. The eSpCas9(1.1), SpCas9-HF1, and HypaCas9 may offer even higher levels of specificity, but are best considered when delivery is performed using plasmid or viral vectors [50–52]. All options should be examined, and which mutant enzyme performs best will likely vary between target sites.

## Conclusions: Best Practices in Mitigating the Risk of Off-Target Editing When Using SpCas9

New genome-editing technologies have the potential to revolutionize treatment of human genetic disorders. When developing new therapeutics, it is always good advice to keep a key phrase from the Hippocratic oath in mind, “first do no harm.” All methods that lead to permanent alterations in genomic DNA have some level of risk to cause changes at off-target sites, which are not meant to be altered. A necessary step in establishing genome-editing protocols is to define what off-target sites are at risk for alteration for each site targeted and take steps to minimize the risk from such events.

The first step in OTE mitigation is to establish the spectrum of sites at risk for a given target site. The sophistication of algorithms that predict off-target sites is rapidly improving; however, such computational tools do not yet have the necessary sensitivity and specificity to stand alone. Bioinformatics prescreening can eliminate obvious risky gRNAs that cross-react with repetitive sites in the genome and can predict some real OTE sites. Nevertheless, all sites of interest should be studied with some experimental method that identifies real OTE events, either *in vitro* or *in vivo*. A set of validated off-target sites should be compiled and used to create a reagent set to quantitatively study OTEs via NGS methods during the research phase where protocols and reagents are optimized. Multiplex amplicon NGS methods are convenient, cost effective, and can be rapidly performed on many samples in parallel. Once assays are in place to monitor OTEs, then the actual genome editing protocols and reagents can be optimized to minimize or eliminate cleavage at the sites at risk.

When possible to use, RNP-based methods provide a simple way to reduce OTE risk by limiting the duration of exposure of the genome to active Cas9 complexes. In some cases, RNP delivery is not feasible and expression-based editing will be necessary, most likely involving viral vectors. Methods have been proposed to use phased temporal expression of CRISPR inhibitors to limit the activity of Cas9 in this setting; however, this approach adds another level of complexity to vector design, which is already stressed to deliver the large Cas9 coding sequence. Using a Cas9 variant that inherently has higher specificity is a far simpler approach.

Engineered Cas9 mutants with improved specificity can significantly reduce OTEs and should be considered for use in all therapeutic genome applications. If using RNP, the “HiFi Cas9” mutant is preferred as this high-fidelity variant retains sufficient cleavage activity to function well in the RNP setting where dose and time are limiting [47]. If using expression-based methods, other mutants might perform better, such as the “eSpCas9(1.1),” “SpCas9-HF1,” or “HypaCas9” variants [50–52]. Today's Cas9 toolbox contains multiple candidates to test for performance in any setting. It is likely that one will work well. In fact, it is hard to imagine any reason to use WT Cas9 today for any precision editing application, especially in translational medical research.
